# Pharmacokinetics, safety and population pharmacokinetics/pharmacodynamics analysis of meropenem-vaborbactam in Chinese healthy participants

**DOI:** 10.3389/fphar.2026.1887991

**Published:** 2026-08-07

**Authors:** Wanzhen Li, Qiong Wei, Mengting Chen, Xiaofen Liu, Beining Guo, Yuancheng Chen, Qi Zhang, Wei Guan, Long Shen, Xiaohui Wang, Nanyang Li, Yan Chen, Xin Li, Yingying Hu, Jing Zhang, Xiaojie Wu

**Affiliations:** 1 Institute of Antibiotics, Key Laboratory of Clinical Pharmacology of Antibiotics, National Clinical Research Center for Aging and Medicine, Joint Laboratory of Hospital & Enterprise for Pathogen Diagnosis of Drug-resistant Bacterial Infections and Innovative Drug R&D, Huashan Hospital, Fudan University, Shanghai, China; 2 Professional Technical Service Platform for Comprehensive Preclinical Pharmacodynamic Evaluation and Clinical Translation of Antibacterial Agents, Huashan Hospital, Fudan University, Shanghai, China; 3 Clinical Pharmacology Research Center, Huashan Hospital, Fudan University, Shanghai, China; 4 SciClone Pharmaceuticals (China) Co., Ltd., Shanghai, China

**Keywords:** meropenem-vaborbactam, Monte Carlo simulation, PK, PK/PD analysis, β-lactamase inhibitor

## Abstract

**Background:**

Meropenem-vaborbactam is a fixed-dose β-lactam/β-lactamase inhibitor combination active against carbapenem-resistant *Enterobacterales* (CRE), especially *Klebsiella pneumoniae* carbapenemase (KPC)-producing CRE. This study evaluated its pharmacokinetics (PK), safety, and tolerability in Chinese participants, and performed population pharmacokinetics/pharmacodynamics (PK/PD) analysis using *in vitro* data to assess the suitability of the standard dosing regimen for treating CRE infections in China.

**Methods:**

This single-center, open-label study included 20 healthy participants divided into two groups receiving meropenem-vaborbactam (1:1) at 1 g/1 g or 2 g/2 g via 3-h infusion. Plasma and urine concentrations of meropenem, its open-ring metabolite, and vaborbactam were analyzed using a validated liquid chromatography-tandem mass spectrometry method. PK analysis was conducted using WinNonlin. Monte Carlo simulations were used to evaluate the 1 g/1 g and 2 g/2 g regimens administered every 8 h as 3-h intravenous infusions. For the standard 2 g/2 g regimen, the impact of extending the infusion duration from 3 to 4 h was further evaluated based on the FDA-recommended PK/PD target.

**Results:**

Plasma concentrations of meropenem, its open-ring metabolite, and vaborbactam peaked immediately after infusion, with rapid distribution and elimination. Exposure increased proportionally with dose, and almost no accumulation was observed after repeated 8-hourly dosing. Most meropenem (68.5%–77.0%) and nearly all vaborbactam were excreted unchanged in urine. Both single and multiple doses were well tolerated, with no severe or serious adverse events. In addition, PK/PD analysis showed that, for CRE isolates, the standard 2 g/2 g every 8 h regimen with a 3-h intravenous infusion achieved ≥90% probability of target attainment for meropenem (45% *f*T > MIC) at MIC values up to 8 mg/L. The corresponding cumulative fraction of response ranged from 98.67 to 100% across ESBL-producing and KPC-producing *Enterobacterales*, including *bla*_KPC_ variants resistant to ceftazidime-avibactam.

**Conclusion:**

The standard meropenem-vaborbactam regimen (2 g/2 g every 8 h, 3-h intravenous infusion) demonstrated favorable PK and safety profiles, with PK/PD analyses supporting the appropriateness of the standard regimen in Chinese participants, consistent with global findings. These results provide PK/PD support for the standard regimen and warrant further evaluation in Chinese patients with CRE infections.

## 1 Introduction

The global detection rate of carbapenem-resistant *Enterobacterales* (CRE) is steadily increasing. According to the results of Antimicrobial Resistance Surveillance System in China (https://www.chinets.com), *Escherichia coli* was the most frequently isolated pathogen from clinical specimens, followed by *Klebsiella pneumoniae*. From 2005 to 2024, resistance rates of *K. pneumoniae* to imipenem and meropenem increased significantly, from 3.0% and 2.9% in 2005 to 22.6% and 23.4% in 2024, respectively. Of particular concern is the recent emergence of a novel subtype of KPC-producing carbapenem-resistant *K. pneumoniae* that exhibits resistance to ceftazidime–avibactam, posing greater challenges to clinical management. These developments underscore the urgent need for new antibacterial agents targeting CRE to address the growing challenge of multidrug-resistant infections.

Meropenem-vaborbactam is a β-lactam/β-lactamase inhibitor combination composed of meropenem, a carbapenem antibiotic, and vaborbactam, a boronic acid–based β-lactamase inhibitor (Bassetti et al., 2022). Meropenem exhibits high stability against extended-spectrum beta-lactamases (ESBL) commonly produced by clinical *Enterobacterales* isolates but is susceptible to hydrolysis by class A serine carbapenemases, such as *Klebsiella pneumoniae* carbapenemase (KPC). Although vaborbactam lacks intrinsic antibacterial activity, it effectively protects meropenem from degradation by these enzymes (Yahav et al., 2020). The fixed-dose formulation (1:1 ratio) and the recommended dosage of 2 g/2 g every 8 h was approved by the U.S. Food and Drug Administration (FDA) in 2017 for the treatment of complicated urinary tract infections (cUTIs), including pyelonephritis, caused by *E. coli*, *K. pneumoniae*, and *Enterobacter cloacae*. In 2018, the European Medicines Agency (EMA) approved for its use in the treatment of cUTIs, complicated intra-abdominal infections, hospital-acquired pneumonia, including ventilator-associated pneumonia, and bloodstream infections that are either confirmed or suspected to be associated with these infection sites.

Meropenem is a time-dependent antibiotic, and its efficacy is best predicted by the percentage of the dosing interval that free drug concentrations remain above the minimum inhibitory concentration (%*f*T > MIC). The pharmacokinetic/pharmacodynamic (PK/PD) target for meropenem typically ranges from 40% to 100% *f*T > MIC (Abdul-Aziz et al., 2020; Bhavnani et al., 2022). For vaborbactam, the primary PK/PD index is the ratio of the 24 h (h) free drug area under the concentration–time curve to the MIC (*f*AUC_0-24_/MIC) (Bhavnani et al., 2022). An *f*AUC_0-24_/MIC ratio of 18–38 correlates with a 1-log bacterial kill. Ratios exceeding 24 have been shown to suppress resistance emergence *in vitro* hollow-fiber infection models (Griffith et al., 2018).

To date, meropenem-vaborbactam has not yet been introduced in China, and its pharmacokinetic (PK) profile, safety, and tolerability in the Chinese population remain unclear. Additionally, geographic variation in pathogen distribution and resistance profiles necessitates region-specific PK/PD evaluations. Integrating local pharmacokinetic data with *in vitro* susceptibility information can help optimize dosing regimens and support evidence-based use of meropenem-vaborbactam in Chinese clinical practice. In this study, we evaluated the safety and PK profiles of meropenem-vaborbactam at 1 g/1 g and 2 g/2 g doses following single and multiple administrations in healthy Chinese participants. In addition, a population PK/PD analysis was performed. By calculating the probability of target attainment (PTA) and cumulative fraction of response (CFR) against various Gram-negative bacteria, this study supports the clinical use of conventional doses and informs the optimization of meropenem-vaborbactam dosing strategies.

## 2 Methods

### 2.1 Participants

A total of 20 healthy participants (male: female = 1:1) aged 18–45 years (inclusive) with a body mass index of 19–28 kg/m^2^ (inclusive) were enrolled in this study. Baseline hematological and clinical biochemical laboratory parameters were within normal limits for all participants. Participants were excluded if they had a known allergy to β-lactam antibiotics, β-lactamase inhibitors, or components of the study drug; a history of allergic diseases; significant systemic illness that could affect drug pharmacokinetics; recent infections or surgeries; history of malignancy; abnormal ECG (QTcF ≥450 ms for males or ≥470 ms for females); impaired renal function (eGFR <90 mL/min); recent use of medications, drug or alcohol abuse, or participation in another clinical trial within 90 days. Participants with positive screening for HIV, hepatitis B or C, or syphilis, as well as those who were pregnant or lactating, were also excluded.

### 2.2 Study designs

This was a single-center, open-label study with single- and multiple-dose administration. Eligible participants were assigned randomization numbers in ascending order of screening number according to a predefined randomization schedule and randomized in a 1:1 ratio to receive meropenem-vaborbactam (1:1) at either 1 g/1 g or 2 g/2 g via 3-h infusions. Each group included 10 healthy participants (5 males and 5 females), totaling 20 participants. On Day 1 (first dose), participants received a single intravenous infusion of meropenem-vaborbactam. From Days 2 – 4, meropenem-vaborbactam was administered via intravenous infusion every 8 h. On Day 5, a single final dose was administered. Plasma and urine samples were collected from all participants within 24 h before and after administration of the first and last doses. In addition, plasma samples for measuring trough and peak concentrations were collected before and after the first dose on Days 3 and 4. The study was performed at the Phase I Unit of Huashan Hospital, China, in accordance with the principles of the Declaration of Helsinki (as revised in 2013). The research protocol and informed consent form were reviewed and approved by the Ethics Committee of Huashan Hospital, Fudan University (No. 2023-478) (clinical trial registration number: CTR20243815). All participants provided written informed consent prior to participation.

### 2.3 Safety and tolerability

All 20 participants were included in the safety analysis. Safety was assessed based on treatment emergent adverse event (TEAE), physical examination, vital signs, and safety laboratory tests, such as hematological tests, urinalysis, serum biochemical tests, and a 12-lead ECG.

### 2.4 Bioanalytical assay

The concentrations of meropenem, its open-ring metabolite, and vaborbactam were quantified using validated liquid chromatography-tandem mass spectrometry (LC-MS/MS) assay following protein precipitation. Chromatographic separation was performed on a polar-modified T3 column, with detection achieved using electrospray ionization (ESI) in positive mode. Meropenem and vaborbactam were analyzed simultaneously, while the open-ring metabolite was measured in a separate assay. The transition ions were *m/z* 384.3 → 141.1 for meropenem, *m/z* 279.9 → 220.1 for vaborbactam, and *m/z* 402.1 → 216.0 for the open-ring metabolite. The assay was validated over concentration ranges of 0.05–50 mg/L for meropenem and vaborbactam in both plasma and urine, and 0.05–15 mg/L in plasma and 0.05–50 mg/L in urine for the open-ring metabolite. Quality control (QC) samples at three or four concentration levels (low, medium, high, and geometric mean concentrations where applicable) were included in each analytical run to ensure assay reliability.

### 2.5 PK study

The specific time points for blood and urine sample collection are shown in Table 1. Plasma PK parameters of meropenem, its open-ring metabolite, and vaborbactam were estimated using non-compartmental analysis with Phoenix WinNonlin software (version 8.3.1). All PK parameters were calculated based on actual time point.

**TABLE 1:** Schedule of blood and urine sampling for pharmacokinetic analysis.

Study day	Dosing	Blood sampling time	Urine sampling time
Day 1	Single-dose	Predose (−2–0 h); 1.5 h, 3 h, 3 h 10 min, 3 h 20 min, 3.5 h, 3 h 45 min, 4 h, 5 h, 6 h, 7 h, 8 h, 12 h, and 24 h after the start of infusion	Predose (−12–0 h); 0–4 h, 4–8 h, 8–12 h, and 12–24 h after the start of infusion
Days 3–4	Multi-dose	Predose (0 h) and 3 h after the end of the first infusions administered each day	NA
Day 5	Multi-dose	Predose (−10–0 min); 1.5 h, 3 h, 3 h 10 min, 3 h 20 min, 3.5 h, 3 h 45 min, 4 h, 5 h, 6 h, 7 h, 8 h, 12 h, and 24 h after the start of infusion	Predose (−2–0 h); 0–4 h, 4–8 h, 8–12 h, and 12–24 h after the start of infusion

NA, not applicable.

### 2.6 Population PK analysis

Population pharmacokinetic (PopPK) analysis was performed using NONMEM (version 7.5.0, ICON Development Solutions, Ellicott City, MD, United States). PsN (version 5.2.6, Uppsala University, Sweden) was used to facilitate model building procedures and to perform covariate analysis and simulations. We evaluated one-, two-, and three-compartment structural models for both meropenem and vaborbactam based on changes in objective function value (OFV) and diagnostic goodness-of-fit (GOF) plots. The final PopPK models for meropenem and vaborbactam were two-compartment models with zero-order input and linear elimination (Trang et al., 2021).

Covariate screening was performed by forward inclusion followed by backward elimination. A covariate was retained if it reached statistical significance at P < 0.01 in both steps. We tested the following covariates for their potential influence on the PK parameters of meropenem and vaborbactam: age, body weight, height, body mass index, body surface area, albumin, alkaline phosphatase, alanine aminotransferase, aspartate aminotransferase, creatinine clearance (CLCr), estimated glomerular filtration rate (eGFR), serum creatinine, hemoglobin, total bilirubin, total protein, hematocrit, and sex. The final model was evaluated using GOF diagnostics and was further validated through visual predictive checks (VPCs) and bootstrap resampling.

To evaluate the impact of covariates on model-predicted exposure, a covariate sensitivity analysis was performed using individual pharmacokinetic parameters estimated from the final PopPK models. Steady-state plasma exposure metrics (AUC, C_avg_, C_max_, and C_min_) were simulated during the final dosing interval following meropenem-vaborbactam 2 g/2 g q8h administered as a 3-h infusion for 5 consecutive days. Continuous covariates (CRCL, eGFR, and WT) were stratified by their median values, and exposure metrics in each subgroup were compared with the median exposure of the overall study population.

### 2.7 PK/PD analysis

The PK/PD index of meropenem is the percentage of the dosing interval during which free plasma concentrations exceed the minimum inhibitory concentration (%*f*T > MIC). Target values of 30%, 35%, 40%, 45%, and 50% were evaluated in this study. For vaborbactam, PK/PD index is defined as the ratio of the area under the free drug plasma concentration-time curve to the minimum inhibitory concentration (*f*AUC_0-24_/MIC), and the target value is *f*AUC_0-24_/MIC ratios of 24 and 38 were evaluated as PK/PD targets. The free fractions of meropenem and vaborbactam were calculated based on plasma protein binding rates of 2% and 33%, respectively (Yahav et al., 2020). PK/PD analyses were performed for the 1 g/1 g and 2 g/2 g regimens administered every 8 h as 3-h infusions. In addition, based on the FDA-recommended meropenem PK/PD target of 45% *f*T > MIC, further analyses were conducted for the standard 2 g/2 g regimen administered every 8 h as a 3-h infusion and the prolonged 4-h infusion regimen.

Using typical values and inter-individual variability estimated from an established PopPK model in healthy Chinese participants, together with MIC distributions from two nationwide *in vitro* susceptibility studies conducted in China, Monte Carlo simulations with 1,000 virtual participants were performed using R software (version 4.3.2) to estimate the PTA and CFR. In general, a PTA or CFR ≥90% was considered indicative of an effective dosing regimen.

In the meropenem-vaborbactam fixed-dose combination, meropenem provides the antibacterial activity, whereas vaborbactam has no intrinsic antibacterial effect. Therefore, the primary PK/PD analyses focused on the probability of achieving pharmacodynamic targets for meropenem against the target pathogens, including *Enterobacterales*, ESBL-producing *Enterobacterales*, and KPC-producing *Enterobacterales* to characterize the antibacterial efficacy of the combination. In parallel, target attainment analyses for vaborbactam were performed to ensure adequate β-lactamase inhibitor exposure across the evaluated dosing regimens.

The two MIC datasets were generated using the CLSI broth microdilution method. Study 1 included 510 *Enterobacterales* clinical isolates collected between 2016 and 2019, primarily comprising *Escherichia coli* and *Klebsiella pneumoniae*, including ESBL-producing and carbapenemase-producing isolates. The MIC_50_ and MIC_90_ values were 0.03 mg/L and 16 mg/L, respectively. Study 2 included 701 *Enterobacterales* clinical isolates collected between 2018 and 2020, including ESBL-producing, KPC-2-producing, NDM-producing, and OXA-48-like-producing isolates. This dataset also contained 18 KPC-2 variant-producing *K. pneumoniae* isolates. The MIC_50_ and MIC_90_ values were 0.5 mg/L and 64 mg/L, respectively, whereas the corresponding values for the KPC-2 variant-producing *K. pneumoniae* subset were 1 mg/L and 4 mg/L.

## 3 Results

### 3.1 Participant demographic and baseline characteristics

A total of 20 eligible healthy participants (10 participants in each group, each with an equal number of males and females) were enrolled and completed the study. The demographic and baseline characteristics of participants are summarized in Table 2.

**TABLE 2:** Participants baseline characteristics.

Characteristics	1 g/1 g (N = 10)	2 g/2 g (N = 10)	Total (N = 20)
Sex, %			
Male	5 (50.0%)	5 (50.0%)	10 (50.0%)
Female	5 (50.0%)	5 (50.0%)	10 (50.0%)
Age, y			
Mean (SD)	35.1 (4.75)	31.4 (4.50)	33.3 (4.89)
Median (range)	34.0 (28–43)	32.0 (26–40)	33.0 (26–43)
Race, %			
Asian, Chinese	10 (100.0)	10 (100.0)	20 (100.0)
Height, cm			
Mean (SD)	162.01 (8.681)	163.84 (7.376)	162.93 (7.896)
Median (range)	162.80 (148.7–174.9)	166.10 (145.8–170.3)	165.55 (145.8, 174.9)
Weight, kg			
Mean (SD)	63.77 (8.211)	63.72 (6.670)	63.75 (7.281)
Median (range)	66.95 (50.0–73.2)	65.45 (54.6–71.4)	65.60 (50.0–73.2)
BMI, kg/m^2^			
Mean (SD)	24.204 (1.3850)	23.743 (2.0207)	23.974 (1.7026)
Median (range)	23.982 (21.95–26.68)	24.572 (20.20–25.97)	24.261 (20.20–26.68)

SD, standard deviation; BMI, body mass index.

### 3.2 Bioanalytical assay

The results of accuracy and precision for the lower limit of quantification and all QC levels are presented in Supplementary Table S1. In plasma, the intra- and inter-batch accuracy ranged from 92.7% to 105.6%, and the corresponding precision ranged from 0.8% to 10.9% for meropenem, its open-ring metabolite, and vaborbactam. In urine, the intra- and inter-batch accuracy ranged from 93.2% to 104.7%, and the corresponding precision ranged from 0.6% to 11.2% for meropenem, its open-ring metabolite, and vaborbactam temperature for up to 6 h, and in urine for up to 4 h. Additional stability data for meropenem and vaborbactam in plasma and urine are provided in Supplementary Table S2.

### 3.3 Safety and tolerability

All 20 participants were included in the safety analysis, and no serious TEAE were reported (Table 3; Supplementary Table S3). Overall, 6 subjects (30.0%) experienced a total of 7 TEAEs. By system organ class (SOC), the most frequently reported events were General Disorders and Administration Site Conditions [5 subjects (25.0%)], followed by Investigations and Gastrointestinal Disorders [1 subject each (5.0%)]. By preferred term (PT), Injection Site Reaction was the most common TEAE, occurring in 5 subjects (25.0%), while Blood Uric Acid Increased and Nausea were each reported in 1 subject (5.0%). One drug-related TEAE was observed in the 1 g/1 g group, identified as an Injection Site Reaction (5.0%). In the 2 g/2 g group, five drug-related TEAEs were reported in four participants, including four Injection Site Reactions (20.0%) and one Nausea event (5.0%). All TEAEs were Grade 1 or 2 in severity, required no pharmacological treatment, and resolved completely.

**TABLE 3:** Drug-related TEAEs in all participants administered meropenem-vaborbactam.

System Organ Class (SOC)	1 g/1 g (N = 10)		2 g/2 g (N = 10)		Total (N = 20)	
Preferred Term (PT)	Subjects (%)	Events	Subjects (%)	Events	Subjects (%)	Events
**Drug-related TEAEs**	1 (10.0)	1	4 (40.0)	5	5 (25.0)	6
**General disorders and administration site conditions**	1 (10.0)	1	4 (40.0)	4	5 (25.0)	5
Injection site reaction	1 (10.0)	1	4 (40.0)	4	5 (25.0)	5
** Gastrointestinal disorders**	0	0	1 (10.0)	1	1 (5.0)	1
Nausea	0	0	1 (10.0)	1	1 (5.0)	1

### 3.4 Pharmacokinetic analysis

**FIGURE 1:**
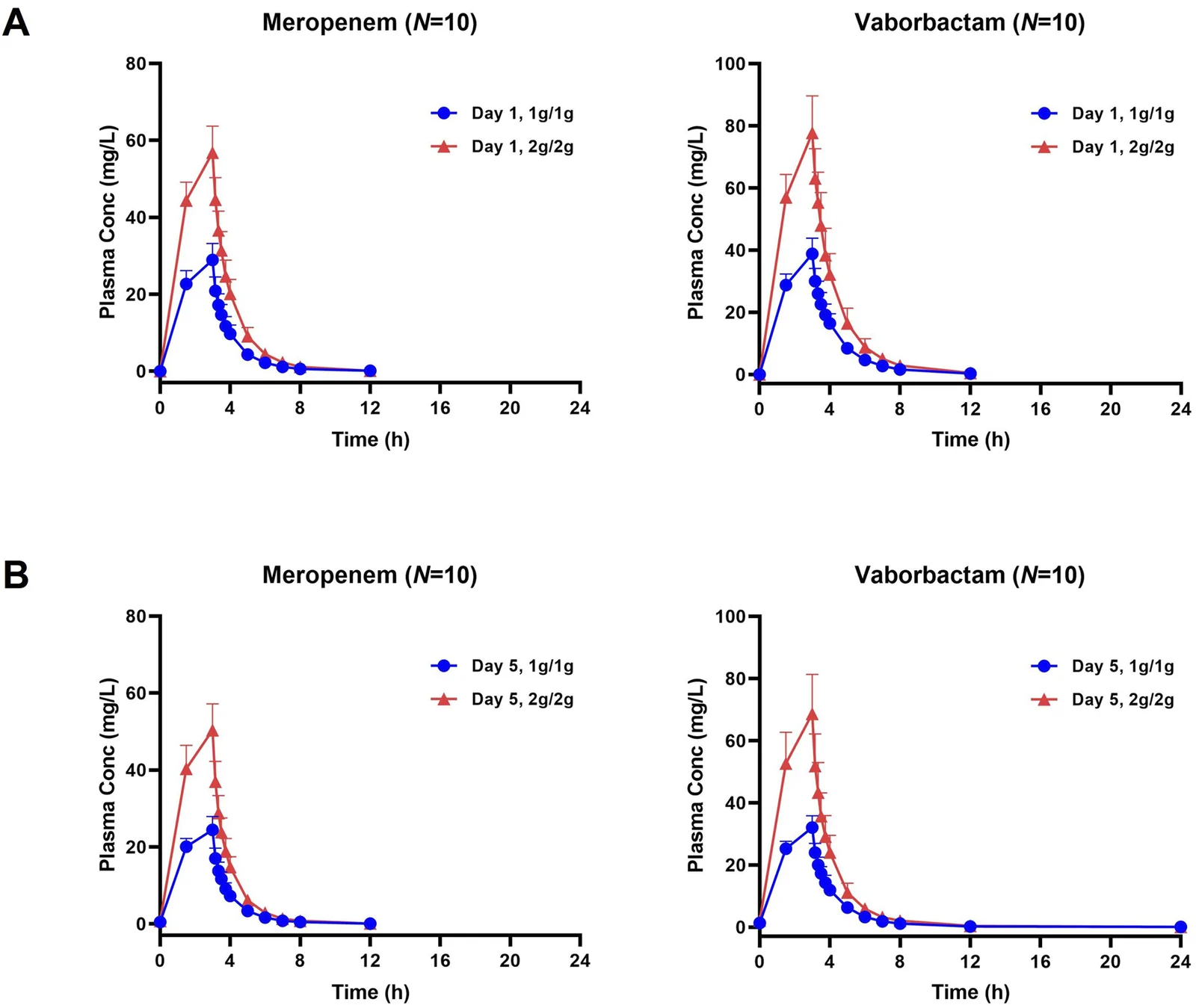
Mean (±SD) plasma total concentration-time profiles of meropenem and vaborbactam. **(A)** on Day 1 following a single 3-h intravenous infusion in 10 participants. **(B)** on Day 5 following multiple 3-h intravenous infusions in 10 participants.

All 20 participants were included in the PK analysis. Figure 1 and Supplementary Figure S1 show the mean plasma concentrations profiles of meropenem, its open-ring metabolite, and vaborbactam following single- and multi-dose intravenous infusion of meropenem-vaborbactam at doses of 1 g/1 g and 2 g/2 g. The corresponding mean plasma PK parameters are summarized in Table 4 and Supplementary Table S4. After single and multiple 3-h infusions, plasma concentrations of meropenem, its open-ring metabolite, and vaborbactam peaked immediately at the end of infusion, followed by rapid distribution and elimination. Exposure parameters, including maximum plasma concentration (C_max_) and area under the concentration–time curve (AUC), increased proportionally with dose. Dose-normalized C_max_ and AUC values were generally comparable between the two dose groups (Supplementary Figure S2). The mean accumulation ratios (AR) between the first and last doses were approximately 0.817–1.09 for all analytes, indicating almost no accumulation with repeated dosing.

**TABLE 4:** Mean plasma PK parameters of meropenem and vaborbactam following single and multiple doses of meropenem-vaborbactam (1 g/1 g and 2 g/2 g) administered via 3-h intravenous infusion.

PK parameters	Meropenem				Vaborbactam			
	Single-dose		Multi-dose^1^		Single-dose		Multi-dose^1^	
	1 g/1 g	2 g/2 g	1 g/1 g	2 g/2 g	1 g/1 g	2 g/2 g	1 g/1 g	2 g/2 g
C_max_, mg/L	28.9 (4.54)	56.8 (7.24)	24.5 (3.64)	50.4 (7.19)	38.8 (5.32)	77.7 (12.5)	32.0 (4.02)	68.6 (13.3)
T_max_, h	2.96 (2.90, 3.05)	2.99 (2.95, 3.10)	2.92 (2.87, 2.95)	2.94 (2.82, 3.02)	2.96 (2.90, 3.05)	2.99 (2.95, 3.10)	2.92 (2.87, 2.95)	2.94 (2.82, 3.02)
AUC_0-τ_, h·mg/L	83.4 (14.3)	168 (22.5)	69.4 (9.76)	140 (21.2)	120 (17.8)	240 (42.0)	97.1 (12.0)	200 (40.7)
AUC_0-inf_, h·mg/L	84.5 (14.8)	170 (22.8)	NA	NA	123 (19.0)	246 (43.5)	NA	NA
T_1/2_, h	1.16 (0.239)	1.20 (0.116)	1.16 (0.230)	1.35 (0.121)	1.52 (0.169)	1.45 (0.141)	1.80 (0.512)	2.54 (0.588)
MRT_0-inf_, h	1.38 (0.117)	1.39 (0.102)	1.31 (0.118)	1.27 (0.0679)	1.72 (0.159)	1.66 (0.161)	1.62 (0.180)	1.56 (0.145)
CL_t_, L/h	12.2 (2.12)	12.0 (1.94)	14.7 (2.01)	14.7 (2.98)	8.30 (1.36)	8.39 (1.76)	10.4 (1.28)	10.5 (2.57)
V_d_, L	19.9 (3.75)	20.8 (3.50)	24.1 (3.32)	28.6 (6.86)	18.1 (2.84)	17.4 (3.51)	26.6 (5.68)	37.2 (7.81)
AR	NA	NA	0.838 (0.0502)	0.833 (0.0607)	NA	NA	0.817 (0.0567)	0.829 (0.0616)
CL_r_, L/h	9.22 (1.74)	8.77 (2.28)	11.3 (1.97)	9.69 (1.47)	9.75 (1.63)	10.2 (2.47)	12.6 (1.62)	11.8 (2.52)
Fe_0-24_, %	76.1 (8.50)	72.7 (9.37)	77.0 (8.13)	68.5 (15.4)	118 (5.66)	121 (8.16)	121 (6.20)	117 (25.2)

Data are expressed as the mean (SD); T_max_ is displayed as Median (Min, Max).

1 The corresponding PK parameters for steady state are C_max, ss_, T_max, ss_, AUC_0-τ, ss_, MRTss, CL_ss_, V_ss_, Fe_0-24, ss_.

C_max_, the peak concentration; AUC_0-τ_, the area under the curve from time 0–8 h; AUC_0-inf_, AUC from time 0 to infinite time; T_1/2_, terminal half-life; CL_t_, total body clearance; V_d_, the volume of distribution; AR, accumulation ratios; CL_r_, renal clearance; Fe_0-24_, cumulative excretion rate from time 0–24 h; NA, not applicable/available.

Only a small fraction of meropenem was metabolized to open-ring metabolite, with metabolic ratios (MR_Cmax,ss_ and MR_AUC,ss_) ranging from 0.107 to 0.173. Within 24 h after the final dose, 68.5%–77.0% of meropenem was excreted unchanged in urine, while 22.4%–24.2% was excreted as the open-ring metabolite. Vaborbactam was minimally metabolized and almost entirely excreted unchanged in urine, accounting for 117%–121% of the administered dose.

### 3.5 Population PK

A total of 570 meropenem and 588 vaborbactam plasma concentrations from 20 subjects were used to develop the PopPK models. For both drugs, the base models were characterized by a two-compartment structure with zero-order input and linear elimination.

For meropenem, time-varying creatinine clearance (CRCL) was included as a covariate on renal clearance in the base model. Following covariate selection, body weight (WT) was retained as a covariate on the peripheral volume of distribution in the final model. The corresponding model equations are shown below.

$$CLNR\;\left(L/hr\right)=3.94$$

$$CLR\;\left(L/hr\right)=10.9\times {CRCL}^{3.51}\;/\;\left(75.5^{3.51}+{CRCL}^{3.51}\right)$$

$$CL\;\left(L/hr\right)=\left(CLNR+CLR\right)\times e^{\eta_{CL,i}}$$

$$Vc\left(L\right)=7.6$$

$$Q\left(L/\text{hr}\right)=6.6$$

$$Vp\left(L\right)=4.77\times {\left(\frac{WT}{65.6}\right)}^{1.07}\times e^{\eta_{Vp,i}}$$

For vaborbactam, time-varying estimated glomerular filtration rate (eGFR) was included as a covariate on renal clearance in the base model. After covariate selection, WT was retained as a covariate on the central volume of distribution in the final model. The corresponding model equations are shown below.

$$CLNR\;\left(L/hr\right)=0.164$$

$$CLR\;\left(L/hr\right)=10.8\times {eGFR}^{3.26}\;/\;\left(73.3^{3.26}+{eGFR}^{3.26}\right)$$

$$CL\;\left(L/hr\right)=\left(CLNR+CLR\right)\times e^{\eta_{CL,i}}$$

$$Vc\left(L\right)=10.3\times {\left(\frac{WT}{65.6}\right)}^{1.51}\times e^{\eta_{Vc,i}}$$

$$Q\left(L/\text{hr}\right)=1.64$$

$$Vp\left(L\right)=2.53$$

In the equations above, CL denotes total clearance from the central compartment, CLNR denotes non-renal clearance from central compartment, CLR denotes renal clearance from the central compartment; Q is the inter-compartment clearance, *V*c is the central volume of distribution, and *V*p is the peripheral volume of distribution. The PopPK model structures for meropenem and vaborbactam are illustrated in Supplementary Figure S3. Parameter estimates from the final PopPK models and bootstrap analyses are summarized in Tables 5, 6, GOF and VPC results are presented in Supplementary Figures S4–S6. Bootstrap analysis demonstrated high model stability, with successful convergence in 100% of runs for meropenem and 99.7% for vaborbactam.

**TABLE 5:** Final meropenem population PK (PopPK) model.

Parameter (Unit)	Original dataset			Bootstrap dataset		Bias (%)
	Estimates (Shrinkage%)	RSE (%)	95% CI	Median	95% CI	
Non-renal clearance from central compartment, CL_NR_ (L/h)	3.94	Fixed	−	3.94	−	−
Central volume of distribution, *V*_C_ (L)	7.60	6.16	6.73–8.57	7.55	6.71–8.53	−0.66
Inter-compartmental clearance, Q (L/h)	6.60	14.3	4.99–8.72	6.60	4.84–8.71	0
Peripheral volume of distribution, *V*_P_ (L)	4.77	8.28	4.06–5.61	4.78	3.99–5.56	0.21
HILL	3.51	24.8	2.18–5.67	3.56	0.924–5.34	1.42
CLCR at 50% of maximal renal clearance, CR_50_ (mL/min)	75.5	4.67	68.9–82.7	77.1	70.3–4220	2.12
Maximal renal clearance, CLRE (L/h)	10.9	9.00	9.17–13.0	11.0	9.63–204	0.92
Effect of body weight on *V*_P_	1.07	14.8	0.761–1.38	1.10	0.787–1.51	2.80
Interindividual variation of CL (%)	8.12 [(1E-10)%]	22.4	2.83–11.1	7.89	4.46–11.4	−2.83
Interindividual variation of *V*_P_ (%)	7.48 (14.6%)	18.9	3.82–9.87	7.08	3.34–9.63	−5.35
Proportional residual error (%)	10.8 (2.9%)	12.7	7.65–13.2	10.4	7.92–13.3	−3.70
Additive residual error (mg/L)	0.0955 (2.9%)	21.4	0.0383–0.129	0.0943	0.0635–0.160	−1.26

RSE, relative standard error; CI, confidence interval.

Bias (%) = (Parameter_Bootstrap dataset_/Parameter_Original dataset_ – 1) × 100%.

**TABLE 6:** Final vaborbactam population PK (PopPK) model.

Parameter (Unit)	Original dataset			Bootstrap dataset		Bias (%)
	Estimates (Shrinkage%)	RSE (%)	95% CI	Median	95% CI	
Non-renal clearance from central compartment, CL_NR_ (L/h)	0.164	Fixed	−	0.164	−	−
Central volume of distribution, *V*_C_ (L)	10.3	4.13	9.53–11.2	10.2	9.08-11.0	−0.97
Inter-compartmental clearance, Q (L/h)	1.64	18.3	1.15–2.34	1.69	1.21–2.75	3.05
Peripheral volume of distribution, *V*_P_ (L)	2.53	10.4	2.07–3.10	2.58	2.12–3.23	1.98
HILL	3.26	21.6	2.15–4.96	3.29	0.876–4.57	0.92
eGFR at 50% of maximal renal clearance, CR_50_ (mL/min)	73.3	3.83	68.0–79.0	74.8	68.4–285	2.05
Maximal renal clearance, CLRE (L/h)	10.8	5.67	9.68–12.1	10.9	9.84–27.7	0.93
Effect of body weight on *V*_C_	1.51	15.9	1.04–1.98	1.54	1.12–2.08	1.99
Interindividual variation of CL (%)	11.5 [(1E-10)%]	19.7	5.49–15.4	11.2	7.00–15.7	−2.61
Interindividual variation of *V*_C_ (%)	7.67 (26.6%)	25.0	1.04–10.8	7.03	0.000667–10.7	−8.34
Proportional residual error (%)	13.1 (2.44%)	7.89	10.9–15.0	12.7	10.6–14.9	−3.05
Additive residual error (mg/L)	0.0689 (2.44%)	20.0	0.0320–0.0920	0.0699	0.0505–0.128	1.45

RSE, relative standard error; CI, confidence interval.

Bias (%) = (Parameter_Bootstrap dataset_/Parameter_Original dataset_ – 1) × 100%.

Covariate sensitivity analyses showed that the effects of body weight and CRCL on meropenem exposure, as well as those of body weight and eGFR on vaborbactam exposure, were generally within −20% to +25% of the population median exposure (Supplementary Tables S5, S6), suggesting a limited influence of these covariates on steady-state exposure within the observed range.

### 3.6 PK/PD analysis

**FIGURE 2:**
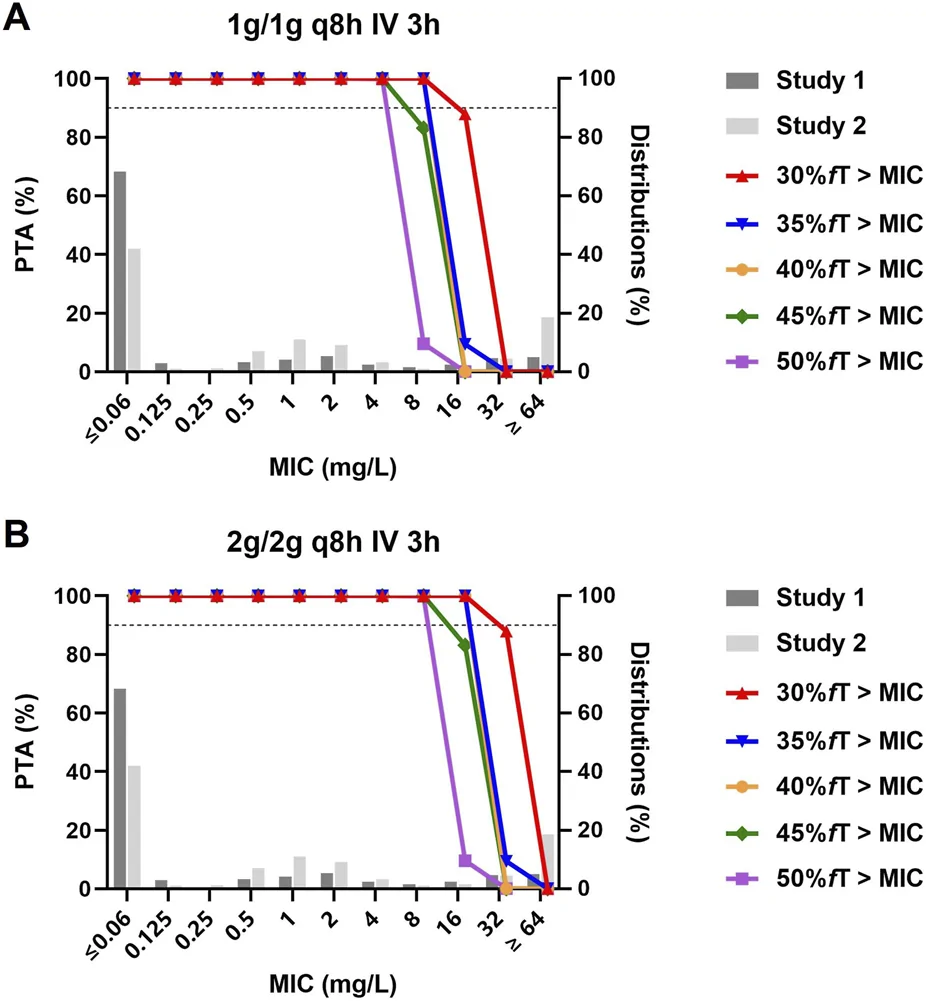
PTA across the MIC distribution for *Enterobacterales* was evaluated under different PK/PD targets and meropenem dosing regimens. The PK/PD targets for meropenem were defined as 30%, 35%, 40%, 45%, and 50% *f*T > MIC. Meropenem-vaborbactam was administered at doses of **(A)** 1/1 g administered as a 3-h IV infusion every 8 h and **(B)** 2/2 g administered as a 3-h IV infusion every 8 h were evaluated. PTA, Probability of Target Attainment; *f*, free fraction; %T > MIC, the percentage of the dosing interval that free drug concentrations remain above the minimum inhibitory concentration.

For a range of meropenem-vaborbactam MIC values and across different PK/PD targets (meropenem %*f*T > MIC of 30%, 35%, 40%, 45%, and 50%), the PTA for the 1 g/1 g and 2 g/2 g regimens administered as 3-h infusions every 8 h is presented in Figure 2. The susceptibility breakpoint for meropenem-vaborbactam was set at 4 mg/L.

For meropenem, under the 1 g/1 g regimen, when MICs were ≤ 8 mg/L, PTA was 100% at PK/PD targets of 30%–40%. At a target of 45% *f*T > MIC, PTA remained 100% for MICs ≤ 4 mg/L but decreased to 83.1% at an MIC of 8 mg/L. At a target of 50% *f*T > MIC, PTA was close to 100% for MICs ≤ 4 mg/L but declined sharply to 9.6% at an MIC of 8 mg/L. For the 2 g/2 g regimen, when MICs were ≤ 16 mg/L, PTA was 100% at PK/PD targets of 30%–40%. At a target of 45% *f*T > MIC, PTA was 100% for MICs ≤ 8 mg/L and 83.1% for an MIC of 16 mg/L. At a target of 50% *f*T > MIC, PTA was close to 100% for MICs ≤ 8 mg/L but decreased to 9.6% at an MIC of 16 mg/L. Overall, the 2 g/2 g standard regimen administered as a 3-h infusion q8h achieved PTAs exceeding 90% across PK/PD targets ranging from 30% to 50% *f*T > MIC for isolates with MICs up to 8 mg/L. The CFR of meropenem under different regimens in plasma are shown in Table 7.

**TABLE 7:** CFR for ESBL-producing and carbapenem-resistant *Enterobacterales* under different dosing regimens across PK/PD targets.

Organism	%*f*T > MIC									
	1 g/1 g					2 g/2 g				
	30%	35%	40%	45%	50%	30%	35%	40%	45%	50%
*In vitro* study 1										
* Enterobacterales* (*N* = 510)	90.11	88.26	88.04	87.77	86.62	94.53	90.83	90.39	89.99	88.26
* *ESBL-producing *Enterobacterales (N* = 65)	100	100	100	100	100	100	100	100	100	100
* *KPC-producing *Enterobacterales* (*N* = 92)	100	100	100	99.63	98.03	100	100	100	100	100
*In vitro* study 2										
* Enterobacterales* (*N* = 701)	76.84	75.61	75.46	75.29	74.56	80.92	77.45	77.03	76.77	75.61
* *ESBL-producing *Enterobacterales (N* = 220)	100	100	100	100	100	100	100	100	100	100
* *KPC-producing *Enterobacterales (N* = 287)	99.22	98.67	98.61	98.55	98.28	99.92	99.37	99.30	99.19	98.67

**FIGURE 3:**
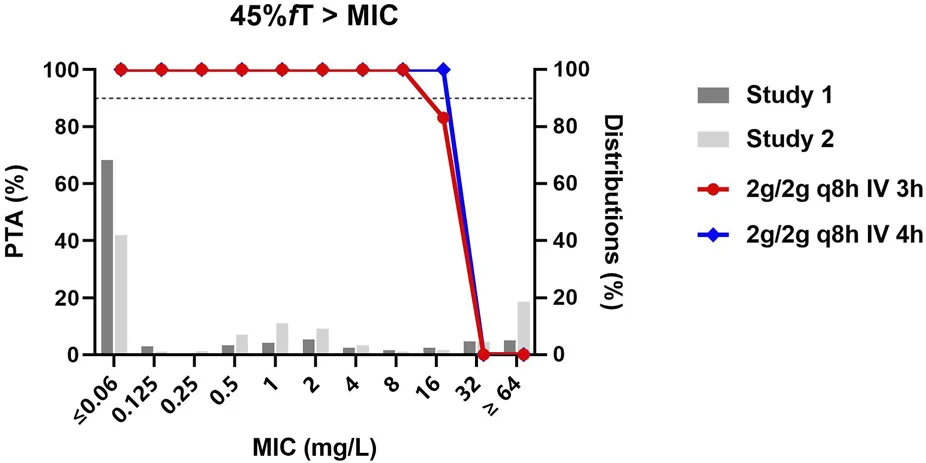
PTA across the MIC distribution for *Enterobacterales* was evaluated at a 45% *f*T > MIC target under meropenem dosing regimens. Meropenem-vaborbactam was administered at doses of 2/2 g as intravenous (IV) infusions over 3 or 4 h every 8 h (q8h). PTA, Probability of Target Attainment; %T > MIC, the percentage of the dosing interval that free drug concentrations remain above the minimum inhibitory concentration. The PK/PD target associated with a 2-log_10_ bacterial reduction was 45% *f*T > MIC, which is also reported for meropenem-vaborbactam in FDA documentation.

Meropenem-vaborbactam was administered at a dose of 2 g/2 g as a 3-h intravenous infusion every 8 h. *In vitro* antibacterial activity study 1, the CFR exceeded 90% at PK/PD targets of 30%–40% *f*T > MIC and remained close to 90% across other target values. In contrast, in study 2, the CFR did not reach 90%, which was likely attributable to the relatively high proportion of resistant isolates (24.53%) with meropenem-vaborbactam MICs ≥16 mg/L, as defined by CLSI breakpoints (CLSI, 2026). However, when the analysis was restricted to isolates with MICs <16 mg/L, PTA values exceeded 90% across the PK/PD target range of 30%–50%. For ESBL- and KPC-producing *Enterobacterales*, CFR values exceeded 90% across PK/PD targets of 30%–50% in both *in vitro* studies. Collectively, these findings suggest that the 2 g/2 g dosing regimen provides favorable antibacterial activity against susceptible *Enterobacterales* isolates. In these simulations, PTA was evaluated at the FDA-reported PK/PD target of 45% *f*T > MIC. The results are shown in Figure 3. At the PK/PD target of 45% *f*T > MIC, all dosing regimens achieved a PTA of 100% for MIC values ≤ 8 mg/L. Extending the infusion duration from 3 h to 4 h with the 2 g/2 g regimen increased PTA at higher MIC values, extending the PTA target attainment threshold from an MIC of 8 mg/L to 16 mg/L at the same total daily dose.

For vaborbactam (Supplementary Figure S7), the PTA was evaluated across a range of MIC values at two PK/PD targets (*f*AUC_0-24_/MIC = 24 and 38). Although vaborbactam lacks intrinsic antibacterial activity, adequate systemic exposure is essential for effective β-lactamase inhibition in combination with meropenem. At the target of *f*AUC_0-24_/MIC = 24, the 1 g/1 g q8h regimen achieved PTA ≥ 90% at MICs up to 8 mg/L, whereas the 2 g/2 g q8h regimen extended this threshold to 16 mg/L. When applying the more stringent target (*f*AUC_0-24_/MIC = 38), the 1 g/1 g q8h regimen-maintained PTA ≥ 90% only at MICs ≤ 4 mg/L, while the 2 g/2 g q8h regimen achieved PTA ≥ 90% up to 8 mg/L.

## 4 Discussion

As meropenem-vaborbactam has not yet been marketed in China, it is essential to evaluate its PK characteristics, safety, and tolerability in the Chinese population. Moreover, given regional variations in bacterial species distribution and antimicrobial resistance patterns, conducting population PK/PD analyses based on local *in vitro* pharmacodynamic data can offer critical insights to inform clinical application in this setting.

The plasma PK profiles of meropenem, its open-ring metabolite, and vaborbactam in Chinese participants were generally comparable to those observed in foreign populations, with exposure levels increasing proportionally with dose. The majority of meropenem (68.5%–77.0%) was excreted unchanged in urine, while a smaller proportion (22.4%–24.2%) was excreted as its open-ring metabolite. Vaborbactam underwent minimal metabolism and was almost entirely excreted unchanged in urine. Mean urinary recovery exceeded 100% in the present study, a finding that has also been reported previously for vaborbactam (Griffith et al., 2016). Given its predominant renal elimination of vaborbactam, estimates of urinary recovery may be sensitive to cumulative variability associated with urine collection and quantification. These PK characteristics are consistent with findings from previous international studies (Wenzler et al., 2015; Rubino et al., 2018). TEAEs related to the study drug were reported in 5 participants (25.0%), all of which aligned with the known safety profile from international Phase I trials (Zhanel et al., 2018). Common adverse events such as headache, somnolence and diarrhea, which are frequently reported in international studies, were not observed (Kaye et al., 2018; VABOMERE, 2017). These findings suggest that the safety risk and tolerability of meropenem-vaborbactam in healthy Chinese participants are favorable. Overall, the safety profile observed in this study is also consistent with international data.

The PK/PD target for meropenem is typically defined as 40%–100% *f*T > MIC. The PK/PD target of 45% *f*T > MIC used in this study was based on FDA data for meropenem-vaborbactam, the standard regimen of meropenem–vaborbactam (2 g/2 g every 8 h via a 3-h infusion) achieved a PTA ≥90% against CRE with an MIC of 8 mg/L, which exceeds the 2026 CLSI susceptibility breakpoint (susceptible ≤4 mg/L). Population PK/PD analyses further demonstrate that extending the infusion duration from 3 to 4 h significantly improves MIC coverage; specifically, a 4-h infusion of 2 g/2 g every 8 h enables 45% *f*T > MIC target attainment for organisms with MICs up to 16 mg/L. For organisms with higher MICs or in critically ill patients requiring more stringent targets (e.g., 100% *f*T > MIC), further optimization strategies may be necessary (Guilhaumou et al., 2019). In ICU patients, prolonging infusion time or adopting continuous infusion may represent more appropriate approaches (Venuti et al., 2023; Gatti et al., 2024). This is supported by PK/PD analysis of continuous infusion (CI) meropenem-vaborbactam in eight ICU patients with ventilator-associated pneumonia caused by KPC-producing *K. pneumoniae* (Gatti et al., 2023). In this retrospective, single-center study, CI administration achieved optimal PK/PD targets and microbiological eradication in most cases. Furthermore, the stability of meropenem-vaborbactam must be considered. According to the European Summary of Product Characteristics, the chemical and physical stability of meropenem-vaborbactam is maintained for up to 4 h at 25 °C (Vaborem, 2023). However, another study reports that meropenem-vaborbactam in 0.9% sodium chloride PVC infusion bags and elastomeric pumps remains stable at room temperature for up to 12 h (Chen et al., 2020), which may support the feasibility of prolonged or continuous infusion strategies in clinical practice. Notably, this study was conducted in healthy participants with normal renal function and therefore may not fully capture the PK variability observed patients with CRE infections, particularly critically ill patients with altered volume of distribution, augmented renal clearance, renal impairment, or hypoalbuminemia. Consequently, further studies are warranted to evaluate dosing optimization under higher PK/PD targets and to confirm stability under real-world clinical conditions.

For vaborbactam, an *f*AUC_0-24_/MIC ratio of ≥18–38 is required to achieve a 1-log kill, while a ratio ≥24 is associated with suppression of resistance (Griffith et al., 2018; Zhanel et al., 2018). The commonly reported PK/PD target of 24, which was chosen based on clinical PK/PD analyses reported in the literature (Gatti et al., 2023; Gatti et al., 2024), is achievable with the approved 2 g/2 g every 8 h via a 3-h infusion regimen, supporting its clinical efficacy against *Enterobacterales* with MICs ≤4 mg/L. Although some case reports have used a target value of 24 in PK/PD analysis for vaborbactam, the optimal PK/PD target for vaborbactam remains to be fully established. Moreover, vaborbactam itself lacks antibacterial activity and is tested at a fixed concentration of 8 mg/L in susceptibility testing. Therefore, in clinical dose optimization, the primary focus should remain on ensuring that meropenem achieves its PK/PD target.

For isolates derived from *in vitro* antibacterial activity study 1, the CFR exceeded 90% when the PK/PD target was set at 30%–40% *f*T > MIC and remained close to 90% at other target values. In contrast, for isolates derived from *in vitro* antibacterial activity study 2, the CFR was below 90%, mainly due to a higher proportion of isolates with meropenem-vaborbactam MICs ≥16 mg/L, which are classified as resistant according to CLSI breakpoints. However, when meropenem-vaborbactam MICs were < 16 mg/L, the PK/PD index achieved PTA values > 90% across target ranges of 30%–50%. In addition, meropenem-vaborbactam demonstrated a PTA of 100% against *bla*_KPC_ variants resistant to ceftazidime-avibactam (Supplementary Table S7), the number of isolates was small (n = 18). To date, over 150 *bla*_KPC_ variants have been reported globally, posing a challenge to clinical treatment (Ding et al., 2023). Given these challenges, there is a need for effective alternative therapies, among which meropenem–vaborbactam has shown promising activity. In a multicenter retrospective study, resistance emerged in three patients with recurrent CRE infections following ceftazidime–avibactam monotherapy, whereas no resistance was observed in those treated with meropenem–vaborbactam (Ackley et al., 2020). Similarly, in a case involving a liver transplant recipient with KPC-producing *K. pneumoniae* bacteremia reported the development of resistance during ceftazidime–avibactam treatment, while alternative regimens failed due to persistent infection or toxicity. Clinical cure was ultimately achieved with meropenem-vaborbactam (Athans et al., 2018). Consistent with these findings, a recently published comparative study of KPC-producing *Enterobacterales* infections also demonstrated a lower frequency of resistance emergence in patients treated with meropenem–vaborbactam than in those receiving ceftazidime–avibactam (0% [0/7] vs. 12% [2/17]) (Karaba et al., 2026). Additionally, monotherapy with meropenem–vaborbactam has been associated with higher clinical cure rates, lower mortality, and reduced nephrotoxicity compared with best available therapy (BAT) for CRE infections (Wunderink et al., 2018). Furthermore, in a clinical study of 59 patients with ceftazidime-avibactam-resistant KPC-producing *K. pneumoniae* infections, 11 patients received meropenem-vaborbactam as salvage therapy (including 9 with meropenem-susceptible and 2 with meropenem-resistant KPC variants). The 30-day mortality rate in the meropenem-vaborbactam group was notably low at 9.1%, compared with 37.5% for carbapenem monotherapy, 15% for non- meropenem-vaborbactam combinations, 30% for colistin-containing regimens, and 75% for tigecycline-containing regimens (Oliva et al., 2023). In a retrospective study, among the subgroup of infections caused by ceftazidime–avibactam-resistant isolates (85, 24.8%), the mortality rate was 24.2% (8/33) in patients who received meropenem-vaborbactam as first-line therapy and 36.5% (19/52) in those who received it as second-line therapy. Notably, the efficacy of meropenem–vaborbactam as a first-line treatment was comparable to that observed in infections caused by ceftazidime–avibactam-susceptible isolates, consistent with findings from previous studies with smaller sample sizes (Tumbarello et al., 2022; Tumbarello et al., 2024). These findings suggest that meropenem-vaborbactam may serve as a viable treatment option in cases of ceftazidime-avibactam resistance.

In conclusion, the standard dosing regimen of meropenem-vaborbactam (2 g/2 g every 8 h via a 3-h intravenous infusion) demonstrates favorable PK characteristics, safety, and tolerability in healthy Chinese participants, consistent with findings from global studies. In critically ill patients or infections caused by organisms with elevated MICs, extending the infusion duration may improve PK/PD target attainment. The results of this study provide PK/PD support for the standard dosing regimen under healthy-volunteer pharmacokinetic conditions and support further evaluation in Chinese patients with CRE infections.

## Data Availability

The data supporting this study’s findings are available from the corresponding authors upon reasonable request. Requests to access the datasets should be directed to Xiaojie Wu.
