# Supplementary material for: Pharmacokinetics, safety and population pharmacokinetics/pharmacodynamics analysis of meropenem-vaborbactam in Chinese healthy participants
Source: Front Pharmacol. 2026 Aug 7;17:1887991. doi: 10.3389/fphar.2026.1887991 (PMC13494463; doi:10.3389/fphar.2026.1887991)
Supplement: Supplementary file 1 [file Supplementaryfile1.docx]

Supplementary Material

# 1 Supplementary Figures and Tables

## 1.1 Supplementary Figures

**Supplementary Figure 1.** Mean (±SD) plasma total concentration-time profiles of open-ring metabolite. (A) on Day 1 following a single 3-h intravenous infusion in 10 participants. (B) on Day 5 following multiple 3-h intravenous infusions in 10 subjects.

**Supplementary Figure 2.** Comparison of dose-normalized PK exposure parameters between the two dose groups following single-dose administration. (A) and (B) show dose-normalized C_max_ and AUC_0–inf_ of meropenem; (C) and (D) show dose-normalized C_max_ and AUC_0–inf_ of vaborbactam. AUC, area under the plasma concentration–time curve; C_max_, maximum plasma concentration; PK, pharmacokinetics. Circles represent individual participant values; stars represent mean values; horizontal lines represent medians. The upper and lower edges of the box indicate the third quartile (Q3) and first quartile (Q1), respectively. The upper and lower whiskers indicate the maximum and minimum values excluding outliers, respectively.

**Supplementary Figure 3.** Schematic diagram of the population PK model for meropenem and vaborbactam. *k*_10_, clearance rate constant from the central compartment; *k*_12_, transfer rate constant from the central to the peripheral compartment; *k*_21_, transfer rate constant from the peripheral to the central compartment; *V*_C_, central volume of distribution; *V*_P_, peripheral volume of distribution.

**Supplementary Figure 4.** Diagnostic plots of the final PopPK model of meropenem. The upper left panel shows observed versus individual predicted concentrations. The upper right panel shows observed versus population predicted concentrations. The lower left panel shows conditional weighted residuals (CWRES) versus time, and the lower right panel shows CWRES versus population predicted concentrations. In all panels, solid black lines indicate reference lines, black dashed lines denote auxiliary boundaries at |CWRES| = 5, and red dashed lines represent locally weighted scatterplot smoothing (LOESS) curves.

**Supplementary Figure 5.** Diagnostic plots of the final PopPK model of vaborbactam. The upper left panel shows observed versus individual predicted concentrations. The upper right panel shows observed versus population predicted concentrations. The lower left panel shows conditional weighted residuals (CWRES) versus time, and the lower right panel shows CWRES versus population predicted concentrations. In all panels, solid black lines indicate reference lines, black dashed lines denote auxiliary boundaries at |CWRES| = 5, and red dashed lines represent locally weighted scatterplot smoothing (LOESS) curves.

**Supplementary Figure 6.** Visual predictive check (VPC) plots for the final population PK model for meropenem (A) and vaborbactam (B). The scattered points represent the prediction-corrected observed plasma concentrations. The red lines represent the median (solid line) and 5th and 95th percentiles (dashed lines) of the observed data. The purple shaded areas represent the 95% confidence intervals of the 5th and 95th percentiles of the prediction-corrected simulated concentrations. The red shaded area represents the 95% confidence intervals of the median of the prediction-corrected simulated concentrations.

**Supplementary Figure 7.** PTA of vaborbactam under different dosing regimens against CRE. PTA, probability of target attainment; *f*, free fraction; MIC, minimal inhibit concentration; AUC_0-24_/MIC, plasma area under the concentration-time curve over 24 hours to the MIC.

## 1.2 Supplementary Tables

**Supplementary Table 1.** Accuracy and precision for meropenem, its open-ring metabolite, and vaborbactam in human plasma and urine.

| **Matrix** | **Analyte** | **Nominal Conc. (mg/L)** | | **Intra-batch (n = 5)** | | | **Inter-batch (n = 15)** | | |
| --- | --- | --- | --- | --- | --- | --- | --- | --- | --- |
|  |  |  |  | **Mean measured Conc. (mg/L)** | **Accuracy (%)** | **CV (%)** | **Mean measured Conc (mg/L)** | **Accuracy (%)** | **CV (%)** |
| Plasma | Meropenem | LLOQ | 0.0500 | 0.0522 | 104.4 | 4.7 | 0.0488 | 97.6 | 9.3 |
|  |  | LQC | 0.150 | 0.147 | 98.0 | 5.3 | 0.148 | 98.7 | 4.6 |
|  |  | GMQC | 1.50 | 1.48 | 98.7 | 2.4 | 1.50 | 100.0 | 3.5 |
|  |  | MQC | 15.0 | 13.9 | 92.7 | 1.4 | 14.7 | 98.0 | 5.2 |
|  |  | HQC | 40.0 | 37.7 | 94.2 | 0.8 | 39.3 | 98.2 | 4.4 |
|  | Vaborbactam | LLOQ | 0.0500 | 0.0485 | 97.0 | 10.9 | 0.0500 | 100.0 | 7.5 |
|  |  | LQC | 0.150 | 0.146 | 97.3 | 2.9 | 0.147 | 98.0 | 3.4 |
|  |  | GMQC | 1.50 | 1.53 | 102.0 | 1.8 | 1.52 | 101.3 | 4.7 |
|  |  | MQC | 15.0 | 15.0 | 100.0 | 1.5 | 14.8 | 98.7 | 6.6 |
|  |  | HQC | 40.0 | 38.5 | 96.2 | 0.8 | 38.3 | 95.7 | 6.4 |
|  | Open-ring metabolite | LLOQ | 0.0500 | 0.0528 | 105.6 | 6.3 | 0.0503 | 100.6 | 8.5 |
|  |  | LQC | 0.150 | 0.150 | 100.0 | 3.9 | 0.146 | 97.3 | 4.9 |
|  |  | MQC | 7.50 | 7.33 | 97.7 | 1.6 | 7.45 | 99.3 | 2.7 |
|  |  | HQC | 12.0 | 11.8 | 98.3 | 1.6 | 11.9 | 99.4 | 2.5 |
| Urine | Meropenem | LLOQ | 0.0500 | 0.0518 | 103.6 | 4.4 | 0.0500 | 100.0 | 8.2 |
|  |  | LQC | 0.150 | 0.148 | 98.7 | 3.4 | 0.152 | 101.3 | 3.5 |
|  |  | GMQC | 1.50 | 1.47 | 98.0 | 1.5 | 1.57 | 104.7 | 5.6 |
|  |  | MQC | 15.0 | 14.3 | 95.3 | 1.9 | 15.2 | 101.3 | 4.9 |
|  |  | HQC | 40.0 | 38.0 | 95.0 | 1.4 | 39.7 | 99.2 | 4.7 |
|  | Vaborbactam | LLOQ | 0.0500 | 0.0466 | 93.2 | 7.7 | 0.0512 | 102.4 | 11.2 |
|  |  | LQC | 0.150 | 0.147 | 98.0 | 4.6 | 0.151 | 100.7 | 6.2 |
|  |  | GMQC | 1.50 | 1.55 | 96.7 | 1.6 | 1.53 | 102.0 | 2.7 |
|  |  | MQC | 15.0 | 14.9 | 99.3 | 0.6 | 14.6 | 97.3 | 2.8 |
|  |  | HQC | 40.0 | 37.6 | 94.0 | 1.8 | 37.5 | 93.7 | 2.7 |
|  | Open-ring metabolite | LLOQ | 0.0500 | 0.0482 | 96.4 | 7.0 | 0.0474 | 94.8 | 5.9 |
|  |  | LQC | 0.150 | 0.155 | 103.3 | 2.4 | 0.153 | 102.0 | 4.1 |
|  |  | GMQC | 1.50 | 1.57 | 104.7 | 1.8 | 1.56 | 104.0 | 1.8 |
|  |  | MQC | 15.0 | 15.1 | 100.7 | 1.3 | 15.2 | 101.3 | 3.3 |
|  |  | HOQ | 40.0 | 39.2 | 98.0 | 2.3 | 39.8 | 99.5 | 2.3 |

LLOC,	lower limit of quantification; QC, quality control; LQC, low concentration QC; GMQC, geometric mean concentration QC; MQC, medium concentration QC; HQC, high concentration QC; CV, coefficient of variation.

**Supplementary Table 2.** The stability of meropenem and vaborbactam in plasma and urine under various conditions.

| **Matrix** | **Analyte** | **Condition** | **LQC** |  | **HQC** | |
| --- | --- | --- | --- | --- | --- | --- |
|  |  |  | **Accuracy (%)** | **CV (%)** | **Accuracy (%)** | **CV (%)** |
| Plasma | Meropenem | Room temperature 6 h | 90.6 | 5.6 | 86.8 | 1.8 |
|  |  | Post-preparation (autosampler,15℃) 48 h | 100.7 | 1.8 | 100.0 | 0.7 |
|  |  | Four freeze-thaw cycles (-70℃) | 104.4 | 6.1 | 100.3 | 3.5 |
|  |  | Long-term 16 days (-20℃)^1^ | 95.3 | 4.4 | 87.4 | 3.0 |
|  |  | Long-term 270 days (-70℃) | 104.7 | 2.8 | 95.3 | 0.7 |
|  | Vaborbactam | Room temperature 6 h | 99.1 | 10.3 | 88.6 | 4.5 |
|  |  | Post-preparation (autosampler,15℃) 48 h | 105.1 | 2.9 | 92.1 | 2.4 |
|  |  | Four freeze-thaw cycles (-70℃) | 101.8 | 3.6 | 98.5 | 0.5 |
|  |  | Long-term 32 days (-20℃) | 98.0 | 3.0 | 91.8 | 2.0 |
|  |  | Long-term 270 days (-70℃) | 102.7 | 5.6 | 97.2 | 1.0 |
| Urine | Meropenem | Room temperature 4 h | 105.6 | 5.1 | 97.7 | 2.0 |
|  |  | 2-8℃ 16h | 103.8 | 5.2 | 96.0 | 2.1 |
|  |  | Post-preparation (autosampler,15℃) 80 h | 108.3 | 3.1 | 98.4 | 2.5 |
|  |  | Four freeze-thaw cycles (-70℃) | 102.9 | 4.6 | 96.3 | 0.4 |
|  |  | Long-term 25 days (-20℃)^2^ | 102.7 | 8.4 | 91.1 | 1.4 |
|  |  | Long-term 93 days (-70℃) | 113.1 | 5.5 | 97.3 | 1.5 |
|  | Vaborbactam | Room temperature 4 h | 103.1 | 5.9 | 93.0 | 2.3 |
|  |  | 2-8℃ 16h | 102.2 | 2.6 | 93.8 | 1.9 |
|  |  | Post-preparation (autosampler,15℃) 80 h | 102.1 | 2.4 | 94.3 | 0.6 |
|  |  | Four freeze-thaw cycles (-70℃) | 101.6 | 3.2 | 92.2 | 1.2 |
|  |  | Long-term 40 days (-20℃) | 90.0 | 5.6 | 88.8 | 1.0 |
|  |  | Long-term 92 days (-70℃) | 106.4 | 5.8 | 94.3 | 2.9 |

QC, quality control; LQC, low concentration QC; HQC, high concentration QC. CV, coefficient of variation.

^1^ A Long-term stability was demonstrated for 16 days at LQC and 32 days at HQC.

^2^ A Long-term stability was demonstrated for 25 days at LQC and 40 days at HQC.

**Supplementary Table 3. S**ummary of TEAEs by SOC and PT

| **System Organ Class（SOC）  Preferred Term（PT）** | **1 g/1 g (N=10)** | | **2 g/2 g (N=10)** | | **Total (N=20)** | |
| --- | --- | --- | --- | --- | --- | --- |
|  | **Subjects（%）** | **Events** | **Subjects（%）** | **Events** | **Subjects（%）** | **Events** |
| **Subjects with at Least One TEAE** | **2 (20.0)** | **2** | **4 (40.0)** | **5** | **6 (30.0)** | **7** |
| **General Disorders and Administration Site Conditions** | **1 (10.0)** | **1** | **4 (40.0)** | **4** | **5 (25.0)** | **5** |
| Injection Site Reaction | 1 (10.0) | 1 | 4 (40.0) | 4 | 5 (25.0) | 5 |
| **Investigations** | **1 (10.0)** | **1** | **0** | **0** | **1 (5.0)** | **1** |
| Blood Uric Acid Increased | 1 (10.0) | 1 | 0 | 0 | 1 (5.0) | 1 |
| **Gastrointestinal Disorders** | **0** | **0** | **1 (10.0)** | **1** | **1 (5.0)** | **1** |
| Nausea | 0 | 0 | 1 (10.0) | 1 | 1 (5.0) | 1 |

**Supplementary Table 4**. Mean plasma PK parameters of open-ring metabolite following single and multiple doses of meropenem- vaborbactam (1 g/1 g and 2 g/2 g) administered via 3-h intravenous infusion.

| **PK parameters** | Open-ring metabolite | | | |
| --- | --- | --- | --- | --- |
|  | **Single-dose** | | **Multi-dose^1^** | |
|  | **1 g/1 g** | **2 g/2 g** | **1 g/1 g** | **2 g/2 g** |
| C_max_, mg/L | 2.81 (0.559) | 6.07 (0.873) | 2.74 (0.448) | 6.22 (0.893) |
| T_max_, h | 2.96 (2.90, 3.05) | 2.99 (2.95, 3.10) | 2.92 (2.87, 2.95) | 2.94 (2.82, 3.02) |
| AUC_0-__τ_, h·mg/L | 10.6 (1.99) | 23.3 (3.86) | 11.2 (1.74) | 25.4 (4.26) |
| AUC_0-inf_, h·mg/L | 12.5 (2.48) | 28.1 (4.82) | NA | NA |
| T_1/2_, h | 2.58 (0.716) | 3.75 (0.666) | 6.65 (1.15) | 6.33 (1.21) |
| MRT_0-inf_, h | 3.49 (0.659) | 4.03 (0.274) | 5.41 (0.647) | 5.32 (0.478) |
| AR | NA | NA | 1.06 (0.0934) | 1.09 (0.0904) |
| CL_r_, L/h | 17.1 (4.01) | 17.8 (3.23) | 21.0 (4.00) | 19.8 (6.06) |
| Fe_0-24_, % | 20.2 (5.69) | 23.6 (6.02) | 22.4 (5.71) | 24.2 (7.90) |

Data are expressed as the mean (SD); T_max_ is displayed as Median (Min, Max).

^1^The corresponding PK parameters for steady state are C_max, ss_, T_max, ss_, AUC_0-τ, ss_, MRT_ss_, Fe_0-24, ss_.

C_max_, the peak concentration; AUC_0-τ_, the area under the curve from time 0 to 8 h; AUC_0-inf_, AUC from time 0 to infinite time; T_1/2_, terminal half-life; AR, accumulation ratios; CL_r_, renal clearance; Fe_0-24_, cumulative excretion rate from time 0 to 24 h; NA, not applicable/available.

Supplementary Table 5. Comparison of meropenem exposure across subgroups stratified by body weight (WT) and creatinine clearance (CRCL)

|  | **WT ≤ Median** | **WT > Median** | **CRCL ≤ Median** | **CRCL > Median** | |
| --- | --- | --- | --- | --- | --- |
| Number of subjects | 10 | 10 | 10 | | 10 |
| WT (kg) | 56.1 (50, 65.1) | 69.9 (66.1, 73.2) | 100.3 (84.9, 104.9) | | 113.6 (106.5, 169.4) |
| AUC_0-τ_ (hr·mg/L) | 161.2 (147.1, 187.8) | 164.8 (120.4, 187.6) | 172 (151.8, 187.8) | | 158.9 (120.4, 178) |
| Difference of AUC_0-τ_ from median (%) | -1.3 | 0.9 | 5.3 | | -2.7 |
| C_avg_ (mg/L) | 20.1 (18.4, 23.5) | 20.6 (15, 23.4) | 21.5 (19, 23.5) | | 19.9 (15, 22.2) |
| Difference of C_avg_ from median (%) | -1.3 | 0.9 | 5.3 | | -2.7 |
| C_max_ (mg/L) | 50.7 (46, 57.6) | 50.3 (38.2, 56.3) | 52.4 (47.3, 57.6) | | 49.6 (38.2, 54.6) |
| Difference of C_max_ from median (%) | 0.5 | -0.2 | 3.9 | | -1.7 |
| C_min_ (mg/L) | 0.7 (0.4, 1.1) | 1 (0.4, 1.6) | 0.9 (0.4, 1.6) | | 0.7 (0.4, 1.4) |
| Difference of C_min_ from median (%) | -20.6 | 17.8 | 13.4 | | -19.7 |

Data are expressed as the Median (Min, Max).

Supplementary Table 6. Comparison of vaborbactam exposure across subgroups stratified by body weight (WT) and estimated glomerular filtration rate (eGFR)

|  | **WT ≤ Median** | **WT > Median** | **eGFR ≤ Median** | **eGFR > Median** |
| --- | --- | --- | --- | --- |
| Number of subjects | 10 | 10 | 10 | 10 |
| WT (kg) | 56.1 (50, 65.1) | 69.9 (66.1, 73.2) | 114.9 (97.9, 118.2) | 129.2 (118.7, 203.9) |
| AUC_0-τ_ (hr·mg/L) | 228.2 (196.8, 272.1) | 209.9 (152.4, 248) | 234.5 (196.2, 272.1) | 214.2 (152.4, 233) |
| Difference of AUC_0-τ_ from median (%) | 6.4 | -2.1 | 9.3 | -0.1 |
| C_avg_ (mg/L) | 28.5 (24.6, 34) | 26.2 (19.1, 31) | 29.3 (24.5, 34) | 26.8 (19.1, 29.1) |
| Difference of C_avg_ from median (%) | 6.4 | -2.1 | 9.3 | -0.1 |
| C_max_ (mg/L) | 67.8 (59.5, 80.3) | 61 (47.5, 69.8) | 68.7 (58.7, 80.3) | 64.2 (47.5, 67.9) |
| Difference of C_max_ from median (%) | 4.5 | -6.1 | 5.8 | -1.1 |
| C_min_ (mg/L) | 2.2 (1.4, 3.3) | 2.4 (0.9, 4.1) | 2.6 (1.7, 4.1) | 2.1 (0.9, 3.8) |
| Difference of C_min_ from median (%) | -11 | 0 | 7.2 | -15.1 |

Data are expressed as the Median (Min, Max).

**Supplementary Table 7.** CFR for KPC variants *Klebsiella pneumoniae* under different dosing regimens across PK/PD targets.

| **Organism** | **%*f*T>MIC** | | | | | | | | | |
| --- | --- | --- | --- | --- | --- | --- | --- | --- | --- | --- |
|  | **1 g/1 g** | | | | | **2 g/2 g** | | | | |
|  | **30%** | **35%** | **40%** | **45%** | **50%** | **30%** | **35%** | **40%** | **45%** | **50%** |
| ***In vitro* study 2** | | | | | | | | | | |
| **KPC variants *Klebsiella pneumoniae* *(N*=18)** | 100 | 100 | 100 | 100 | 99.99 | 100 | 100 | 100 | 100 | 100 |
